# Modelling prevalent cardiovascular disease in an urban Indigenous population

**DOI:** 10.17269/s41997-022-00669-x

**Published:** 2022-08-09

**Authors:** Lisa Avery, Raglan Maddox, Robert Abtan, Octavia Wong, Nooshin Khobzi Rotondi, Stephanie McConkey, Cheryllee Bourgeois, Constance McKnight, Sara Wolfe, Sarah Flicker, Alison Macpherson, Janet Smylie, Michael Rotondi

**Affiliations:** 1 Department of Biostatistics, Princess Margaret Cancer Centre, University Health Network, Toronto, ON Canada; 2 Dalla Lana School of Public Health, University of Toronto, Toronto, ON Canada; 3 Well Living House, Li Ka Shing Knowledge Institute, Unity Health Toronto - St. Michaels Hospital, Toronto, ON Canada; 4 Aboriginal and Torres Strait Islander Health Group, National Centre for Epidemiology and Public Health, Research School of Population Health, The Australian National University, Canberra, Australia; 5 School of Kinesiology and Health Science, York University, Toronto, ON Canada; 6 Ontario Tech University, Oshawa, ON Canada; 7 Seventh Generation Midwives Toronto, Toronto, ON Canada; 8 Metropolitan University, Toronto, ON Canada; 9 De dwa da dehs nye>s Aboriginal Health Centre, Brantford, ON Canada

**Keywords:** Indigenous health, Cardiovascular disease, Respondent-driven sampling, Model validation, Discrimination, Santé autochtone, maladies cardiovasculaires, échantillonnage en fonction des répondants, validation de modèle, discrimination

## Abstract

**Objective:**

Studies have highlighted the inequities between the Indigenous and non-Indigenous populations with respect to the burden of cardiovascular disease and prevalence of predisposing risks resulting from historical and ongoing impacts of colonization. The objective of this study was to investigate factors associated with cardiovascular disease (CVD) within and specific to the Indigenous peoples living in Toronto, Ontario, and to evaluate the reliability and validity of the resulting model in a similar population.

**Methods:**

The Our Health Counts Toronto study measured the baseline health of Indigenous community members living in Toronto, Canada, using respondent-driven sampling. An iterative approach, valuing information from the literature, clinical insight and Indigenous lived experiences, as well as statistical measures was used to evaluate candidate predictors of CVD (self-reported experience of discrimination, ethnic identity, health conditions, income, education, age, gender and body size) prior to multivariable modelling. The resulting model was then validated using a distinct, geographically similar sample of Indigenous people living in Hamilton, Ontario, Canada.

**Results:**

The multivariable model of risk factors associated with prevalent CVD included age, diabetes, hypertension, body mass index and exposure to discrimination. The combined presence of diabetes and hypertension was associated with a greater risk of CVD relative to those with either condition and was the strongest predictor of CVD. Those who reported previous experiences of discrimination were also more likely to have CVD. Further study is needed to determine the effect of body size on risk of CVD in the urban Indigenous population. The final model had good discriminative ability and adequate calibration when applied to the Hamilton sample.

**Conclusion:**

Our modelling identified hypertension, diabetes and exposure to discrimination as factors associated with cardiovascular disease. Discrimination is a modifiable exposure that must be addressed to improve cardiovascular health among Indigenous populations.

**Supplementary Information:**

The online version contains supplementary material available at 10.17269/s41997-022-00669-x.

## Résumé

**Objectif:**

Des études ont souligné les iniquités entre les populations autochtones et non autochtones en ce qui a trait au fardeau des maladies cardiovasculaires et à la prévalence des risques prédisposants qui résultent des impacts historiques et continus de la colonisation. Nous avons voulu étudier les facteurs associés aux maladies cardiovasculaires (MCV) au sein des populations autochtones vivant à Toronto (Ontario) et spécifiques à ces populations, puis évaluer la fiabilité et la validité du modèle ainsi obtenu dans une population semblable.

**Méthode:**

L’étude Our Health Counts Toronto a mesuré l’état de santé de départ de membres de communautés autochtones vivant à Toronto, au Canada, à l’aide d’un échantillonnage en fonction des répondants. Une approche itérative, valorisant à la fois les données d’études scientifiques, l’expérience clinique, le vécu de personnes autochtones et les mesures statistiques, a été employée pour évaluer les candidats prédicteurs des MCV (expérience autodéclarée de discrimination, identité ethnique, affections médicales, revenu, instruction, âge, genre et taille) avant la modélisation multivariée. Le modèle ainsi obtenu a ensuite été validé à l’aide d’un échantillon distinct, mais géographiquement similaire, de personnes autochtones vivant à Hamilton (Ontario), au Canada.

**Résultats:**

Le modèle multivarié des facteurs de risque associés aux MCV prévalentes incluait l’âge, le diabète, l’hypertension artérielle, l’indice de masse corporelle et l’exposition à la discrimination. La présence combinée du diabète et de l’hypertension artérielle était associée à un risque accru de MCV comparativement à l’une ou l’autre de ces deux affections médicales; c’était aussi la plus forte variable prédictive des MCV. Les personnes ayant déclaré des expériences passées de discrimination étaient aussi plus susceptibles d’être atteintes de MCV. D’autres études sont nécessaires pour déterminer l’effet de la taille sur le risque de MCV dans la population autochtone urbaine. Le modèle final avait un bon pouvoir discriminant et une calibration adéquate lorsqu’il a été appliqué à l’échantillon de Hamilton.

**Conclusion:**

Notre modélisation a cerné l’hypertension artérielle, le diabète et l’exposition à la discrimination comme facteurs associés aux maladies cardiovasculaires. La discrimination est un risque modifiable qui doit être abordé pour améliorer la santé cardiovasculaire au sein des populations autochtones.

## Introduction

The high burden of cardiovascular disease (CVD) among Indigenous (First Nations, Inuit and Métis) communities in Canada has been well documented (Anand et al., 2006; Monsalve et al., 2005). Encouragingly, Chu et al. (2019) reported that rates of CVD among First Nations with diabetes living in Ontario decreased from 1996 to 2015, but remained higher than for other people in Ontario (which included Métis and Inuit peoples). It is commonly accepted that race and ethnicity are not biological, but they have tangible and meaningful impacts on Indigenous health outcomes (Watego et al., 2021). Evidence indicates that racialized differences in CVD are not due to genetics and physiology, meaning such inequities are due to other factors (Foulds et al., 2018). Despite evidence that traditional models of CVD risk perform worse for First Nations peoples than for the white or Black populations (D’Agostino et al., 2001), relatively little work has been done to identify risk factors specific to this population. The Study of Health Assessment and Risk Evaluation in Aboriginal Peoples was conducted to investigate the rates of CVD and atherosclerosis and their risk factors among the First Nations population in Canada and to compare risk factors with the general population (Anand et al., 2001). The authors found increased CVD burden among First Nations participants, and increased prevalence of risk factors such as smoking, diabetes and obesity compared to the general population. However, the generalizability of those findings is limited because all First Nations participants lived on a single reserve. Monsalve et al. (2005) compared the distribution of risk factors between those of Nuxalk descent and non-Indigenous community members living in British Columbia and found differences in blood lipid and glucose levels and body mass index but did not determine whether these translated into different rates of CVD. A comprehensive review of CVD risk factors across ethnic groups within North America found that diabetes, obesity and smoking were more prevalent among Indigenous populations than in the ‘white’ population (Gasevic et al., 2015), consistent with Lucero et al. (2014) who reported higher prevalence of CVD risk factors among respective Indigenous (vs. non-Indigenous) populations in Aotearoa New Zealand, Australia and the United States.

Differences in the prevalence of risk factors between the Indigenous and non-Indigenous populations are well established (Anand et al., 2001; Gasevic et al., 2015; Monsalve et al., 2005). The reasons for the disproportionately high prevalence of risk factors among Indigenous peoples are complex, with evidence indicating that the drivers associated with such risk factors are similar for Indigenous and non-Indigenous peoples. However, as a result of colonization these drivers are more common within Indigenous populations. The roots of such causes can be found in historic and current governmental programs and policies that disrupt Indigenous societies, including colonization and active exclusion and erosion of social structures, economies, education system and food supplies (Anand et al., 2019). Now the focus needs to shift to identifying modifiable risk factors for Indigenous peoples and, in particular, the fast-growing urban Indigenous community. Work has begun; a study protocol published by Rémond et al. (2017) aims to uncover risk factors specific to the Indigenous population in Australia and Anand et al. (2019) undertook an ecological study of risk of CVD at the community level. We aim to inform similar work by describing sociodemographic, behavioural and social determinants associated with disease prevalence specific to the urban Indigenous population in Canada.

A common research shortcoming is the narrow definition of Indigenous peoples in Canada which has historically been defined as those with band membership, residence on a reservation, or registered Indian status. The First Nations Regional Longitudinal Health Survey, a valuable source of information on disease prevalence, samples only from registered First Nations living on-reserve (First Nations Information Governance Centre, 2005). As Lavoie et al. (2010) identified, there are difficulties with such research; reserve and Indian status-based studies can no longer adequately describe the health of a population undergoing a rapid transition to urban centres. According to Statistics Canada, the Indigenous population off-reserve is the fastest-growing segment of Canadian society; 56% of Indigenous people live in urban areas and the off-reserve population grew by 49% between 2006 and 2016 (Statistics Canada, 2017). This transition requires a corresponding shift in how health research is conducted for, and with, this population.

Our objective was to explore factors that may increase or reduce risk of CVD among Indigenous people living in Toronto, Canada, using a multi-disciplinary approach to guide our analyses. Our secondary objective was to validate the model using a distinct sample of urban Indigenous community members living in Hamilton, Ontario. This study reports data collected from the Our Health Counts (OHC) Toronto study, in accordance with Strengthening the Reporting of Observational Studies using Respondent-Driven Sampling (STROBE-RDS) guidelines (White et al., 2015).

## Methods

### Research team

OHC Toronto was a collaborative study between Seventh Generation Midwives Toronto (SGMT) and researchers from the Well Living House (WLH). The WLH is an action research centre focused on building and sharing evidence to support Indigenous infant, child and family health and well-being and is co-governed by a Counsel of Indigenous Grandparents and St. Michael’s Hospital. We recognize that the methodology and research team members’ worldviews can influence our perspectives and values (Smith, 2012). In prioritizing the lived experience and worldviews of Indigenous peoples, this work was governed by Indigenous peoples and the research team included Indigenous community members, midwives, a physician-scientist, community leaders, students and allies, including statisticians, an epidemiologist and student researchers. We relied on medical, social and epidemiological experience and expertise relevant to the community, published literature and observed results from OHC Toronto. The project was approved by the WLH and SGMT, who assisted to ensure the safe-guarding of the data. The Knowledge Keeper (SGMT) contributed to, reviewed and approved this manuscript. This project has been reviewed and approved by the Research Ethics Board of St. Michael’s Hospital (REB no: 14-083). A thorough description of the study procedures has been previously reported (Rotondi et al., 2017) and a brief overview is provided here.

### Our Health Counts

The Our Health Counts (OHC) studies are Indigenous-led studies designed to establish baseline health information for the Indigenous populations in Hamilton, London, Ottawa, Toronto, Thunder Bay and Kenora using respondent-driven sampling (RDS) to obtain representative samples. Respondent-driven sampling is a chain-referral snowball sampling technique used to sample hard-to-reach populations when random sampling is not possible. RDS improves on snowball sampling by using the relationships between the number and nature of connections within the sample to obtain asymptotically unbiased estimates of disease prevalence (Heckathorn, 2002). However, Avery et al. (2019) have shown that unadjusted regression analysis is preferable when the goal is to examine factors associated with disease prevalence in RDS samples. RDS was appropriate for the OHC studies given the lack of a sampling framework of Indigenous people in urban centres, and strong community ties building on social and kinship lines of trust and accountability.

### Study participants

Using RDS, the Indigenous community in Toronto was surveyed between March 2015 and March 2016. Participants were interviewed in-person, using a respectful health survey, at three locations providing health and social services. The respectful health survey, as opposed to a rapid health assessment, was administered by individuals with strong pre-existing Indigenous community engagement skills and who were trained to ensure that all interviews were conducted in a respectful and culturally sensitive manner. Recruitment started with ten seeds and three recruitment coupons per participant. After enrolment commenced, an additional ten seeds were recruited, and the number of coupons increased to five per participant to speed recruitment. Eligibility criteria were the following: (1) residing, working or receiving social services in Toronto, (2) identifying as a member of the Indigenous community, and (3) being at least 15 years old. Participants were permitted to participate only once. Duplicates were identified through provincial health card numbers, which 97% of respondents voluntarily provided. Participants received $20 for participating and $10 for each person they recruited. Recruitment chains were traced using unique codes on the recruitment coupons. To measure network degree, participants were asked “Approximately how many Aboriginal people do you know (i.e., by name and that know you by name) who currently live, work or use health and social services in Toronto?”. The model validation work was performed on the OHC Hamilton database, a sample of 554 adults recruited using RDS, in a city approximately 70 km from Toronto. Details of this sample have been reported previously (Firestone et al., 2014).

### Modelling approach

As this was a secondary analysis of cross-sectional data collected to measure baseline health, CVD prevalence was modelled. When describing disease prevalence (as opposed to incidence), risk factors need to be evaluated based on theory and the existing evidence base about the causes of CVD, in addition to the observed data. This required careful deliberation of our initial set of variables to ensure that observed associations were likely causal in nature and that exposures were not influenced by disease status. For example, under the social supports model that exists in Toronto, a diagnosis of CVD could qualify someone for social assistance, thereby directly impacting their social determinants of health. What appears to be a risk factor may instead be the result of disease, and so careful consideration of causal pathways was integral to the modelling process. This was facilitated by the specialist knowledge of the Indigenous community research members who included a midwife, physician-researcher and epidemiologist. Each candidate predictor considered for inclusion had a strong theoretical justification and variables that we expected to be affected by a CVD diagnosis were described and discussed. Model development was undertaken on a sample from Toronto, Ontario, and validated with distinct, but similar, data from Indigenous peoples living in nearby Hamilton, Ontario.

### Statistical methods

To determine whether the sample size was large enough and diverse enough to estimate CVD prevalence, convergence plots were examined (Gile et al., 2015). Once estimates have stabilized across successive waves, the sample has reached equilibrium, indicating adequate sample size.

In studies of disease prevalence using RDS, the sample must be weighted to account for the non-random probability of participant selection. Naive and RDS-adjusted estimates using the RDS-II estimator (Volz & Heckathorn, 2008) of CVD prevalence were calculated. Unweighted Poisson regression was used to estimate the relative risk and was chosen in favour of the Binomial model for two reasons: (1) previous work indicated that type I error was maintained over a broader range of conditions and that the model was generally conservative (Avery et al., 2019), which was important given our many predictor variables; and (2) Poisson regression provides a direct estimate of relative risk (RR), which is more easily interpreted than the odds ratio (OR). Unweighted analyses were performed because of previous findings that unweighted Poisson models have superior validity and coverage rate for RDS data (Avery et al., 2019). All modelling was performed in the R statistical language (R Core Team, 2020), and RDS-adjusted prevalence of CVD was calculated using the RDS package (Handcock et al., 2019). Seeds were excluded from the analyses.

### Variables

The outcome of interest was CVD, scored dichotomously as self-reported diagnosis of stroke, or heart disease by a healthcare professional. Drawing on the Respectful Health Assessment Survey results and existing literature, our multi-disciplinary team comprised of Indigenous epidemiologists, community-situated Indigenous service providers and biostatisticians identified the following predictors for consideration: age, gender, measured BMI, diabetes, hypertension, cigarette smoking, exercise, education, income, housing, Indigenous self-identity and experiences of discrimination. These variables are defined in Table 2 and further background is provided in the Supplemental Information. To determine which variables to include in the multivariable model, we considered the relationship between each variable and risk of CVD, controlling for age, the single most important predictor of CVD (D’Agostino et al., 2001). These relationships were examined (using regression coefficients and data visualization) and discussed to ensure they fit our expectations under a causal model. Further details on the variable selection process are provided in the Supplemental Information. Missing values were treated by case-wise deletion, and missing data are described in Table 1.

**Table 1:** Our health counts Toronto study sample characteristics (*n*=897)

Variable	*N* (%)	Missing, *N* (%)	RDS-adjusted prevalence (95% CI)
Age group		0	
< 40 years	382 (42.6)		51.8 (46.1, 57.5)
40–64 years	458 (51.0)		44.7 (39.0, 50.3)
65 years and older	57 (6.4)		3.5 (0.7, 6.2)
BMI group		26 (2.9)	
Healthy weight	310 (34.6)		40.4 (34.6, 46.1)
Underweight (BMI < 18.5 kg/m^2^)	18 (2.0)		3.2 (1.2, 5.2)
Overweight (BMI > 25 kg/m^2^)	273 (30.4)		30.2 (25.1, 35.3)
Obese I (BMI > 30 kg/m^2^)	157 (17.5)		17.3 (12.4, 22.2)
Obese II (BMI > 35 kg/m^2^)	68 (7.6)		6.0 (3.5, 8.5)
Obese III (BMI > 40 kg/m^2^)	45 (5.0)		2.9 (1.2, 4.6)
Social determinants			
Above before-tax LICO	172 (19.2)	14 (1.6)	12.1 (8.5, 15.8)
Completed high school	503 (56.1)	2 (0.2)	49.6 (43.9, 55.3)
Lives alone	320 (35.7)	3 (0.3)	29.0 (23.7, 34.2)
Married	46 (5.1)	5 (0.6)	4.0 (2.1, 5.9)
Unemployed	474 (52.8)	0 (0.0)	62.3 (56.8, 67.8
Lifestyle			
Current smoker	609 (67.9)	6 (0.7)	63.1 (57.3, 68.9)
Drinking excessively	480 (53.5)	6 (0.7)	47.3 (41.5, 53.0)
Exercise days/week		4 (0.4)	
0 days per week	60 (6.7)		7.1 (4.0, 10.3)
1 day per week	44 (4.9)		3.7 (1.2, 6.2)
2 days per week	58 (6.5)		6.6 (4.1, 9.2)
3 days per week	98 (10.9)		13.3 (9.4, 17.1)
4 days per week	68 (7.6)		8.0 (4.9, 11.1)
5 days per week	76 (8.5)		10.1 (6.7, 13.6)
6 days per week	27 (3.0)		4.3 (2.4, 6.3)
7 days per week	462 (51.5)		46.8 (41.1, 52.4)
Comorbidity			
Diabetes	155 (17.3)	5 (0.6)	15.0 (10.9, 19.1)
High blood pressure	217 (24.2)	8 (0.9)	23.9 (18.8, 29.0)
Discrimination	734 (81.8)		76.8 (71.8, 81.7)
Outcome: cardiovascular disease	106 (11.8)	0	8.9 (5.5, 12.2)

The RDS-adjusted prevalence estimates the population-level distribution of the demographic variables. These estimates adjust for the unequal sampling probability due to the RDS sampling design

#### A multivariable model of CVD

Our initial multivariable model of factors associated with CVD contained variables which appeared to contribute little unique information and so we sought to fit a more parsimonious model. To simplify comparisons across models, we evaluated the multivariable model using a reduced sample (*n*=785) with complete data. A number of statistics were calculated for the full model, and for models removing each variable in turn, these were the following: Akaike’s Information Criteria (AIC), Nagelkerke’s (1991) pseudo *R*^2^ value, model sensitivity, specificity, positive and negative predictive values and accuracy. The predictive statistics were calculated by comparing the number of participants reporting CVD and the number predicted by the model. Variables were removed from the multivariable model if the model fit was improved, as indicated by a lower AIC, and if none of the predictive statistics were made worse. The relative risks for the remaining variables were checked to ensure stability and to detect potential confounding. These steps were repeated until the model fit and prediction could not be further improved. The final model was then estimated for the final set of predictors with all data available for those variables.

#### Multivariable model validation

Due to the observed data informing the analytic choices (as opposed to testing a priori relationships), it was necessary to evaluate the final model to determine if we had over-fit the data, or if the results could be replicated. The predictive ability of our model was validated using a distinct but geographically similar sample of Indigenous peoples from the OHC Hamilton study. The *c*-index was used to assess model discrimination (Harrell & Slaughter, 2001, p. 247). Model calibration was examined for risk deciles and the Hosmer-Lemeshow *χ*^2^ statistic was computed. To account for different CVD prevalence in the samples, a conversion factor was added to the model intercept. The Poisson model equivalent of the conversion factor proposed by Janssen et al. (2009) was computed as $\mathrm{CF}=\ln \left(\frac{{\hat{p}}_{\mathrm{validation}}}{\mathrm{MP}{\mathrm{R}}_{\mathrm{validation}}}\right)$, where $\hat{p}$ is the disease prevalence and MPR is the mean predicted risk. Finally, to describe the relative risk of the model in the Hamilton sample, the model was re-estimated using the Hamilton data.

## Results

### Participants

There were 3505 coupons issued during the study, resulting in 959 recruits in addition to 20 seeds. After removing those ineligible for the study, duplicates and seeds, 897 individuals were retained for analysis. Participants ranged in age from 15 to 80 years, with a mean age of 42.5, and 460 (51.3%) were women, 420 (46.8%) were men, and 17 (1.9%) were gender diverse. Mean sample BMI was 27.8 kg/m^2^ with a low of 16.5 kg/m^2^ and a high of 56.2 kg/m^2^. Sample demographics, including missing data, are included in Table 1. The reported degree approximated a log-normal distribution with median degree of 50, inter-quartile range of 20–150 and mean of 165. Convergence plots (not shown) indicated that prevalence estimates for self-reported CVD were stable after 750 participants were recruited.

### Evaluating candidate predictors

From our original list of 12 candidate predictors, we chose to include: age, gender, measured BMI (categorized as underweight <18.5 kg/m^2^, healthy/overweight 18.5–30 or obese 30+), a meta-variable combining self-reported diabetes and hypertension, income (dichotomously scored as below or above the low-income cutoff), education (dichotomously scored as having achieved a tertiary qualification or not), score on the Multi-Ethnic Identity Measure (MEIM total score), and a dichotomous variable indicating any reports of discrimination; variable details are provided in Table 2 and further background is given in the Supplemental Information. Table 3 presents the preliminary multivariable model.

**Table 2:** Variable coding for candidate predictors of cardiovascular disease

Variable	Details
Age	Age at the time of the survey was calculated from self-reported date of birth
Body mass index	Height and weight were both measured during the survey and BMI was calculated as weight (kg)/ height (m)^2^
Cigarette smoking	Self-reported smoking status (do you presently smoke cigarettes, yes/no)
Diabetes, hypertension	Self-reported diabetes diagnosed by a healthcare provider, self-reported high blood pressure, combined into a single variable with four levels: those with (1) neither condition, (2) both conditions, (3) diabetes only, or (4) hypertension only
Discrimination	Participants in OHC Toronto were asked: if their health/well-being had been affected by racism, if they had been treated poorly or unfairly because of being Aboriginal or by a healthcare worker because of being Aboriginal or if they had been the victim of an ethnically or racially motivated attack, whether discrimination was a source of stress they experienced or whether they had been treated unfairly because of their gender/sexual orientation or due to mental health problems. Participants in OHC Hamilton were asked if they had experienced any of the following: unfair treatment because of being Indigenous, unfair treatment because of mental or emotional problems, unfair treatment because of gender or unfair treatment by a healthcare worker. An affirmative answer to any of the questions was considered exposure to discrimination.
Education	Self-reported highest level of training and/or education dichotomized into tertiary education (defined as all those who completed university, college or specialized trades training) vs no tertiary (having primary or secondary education)
Exercise	Self-reported average days per week of doing 30 min of moderate or hard physical activity
Gender	Self-identified gender
Housing	Self-reported living situation categorized into (1) stable housing (having a permanent place to stay), (2) precarious housing (staying with friends or relatives or in a motel), (3) institutionalized (nursing home or hospital), or (4) homeless
Income	Data on total household income and household size were collected and used, along with tables provided by Statistics Canada (Statistics Canada, 2015) to dichotomize participants into those above and below the before-tax low-income cutoff (LICO)
Indigenous identity	Assessed using the total score from the Multi-Ethnic Identity Measure (MEIM) (Phinney, 1992)

**Table 3:** Relative risk of cardiovascular disease for the candidate variables in the initial multivariable model (n=785)

Variable	RR (95% CI)	*p* value
Age	1.05 (1.03, 1.07)	<0.001
Gender		
Men	1.00 (Reference)	
Women	1.1 (0.72, 1.71)	0.661
Diabetes and hypertension		
Neither condition	1.00 (Reference)	
Diabetes	1.58 (0.69, 3.30)	0.251
Hypertension	2.81 (1.62, 4.91)	<0.001
Both conditions	2.85 (1.55, 5.22)	<0.001
Body mass index (BMI)		
Normal or overweight (18.5-30 kg/m^2^)	1.00 (Reference)	
Underweight (< 18.5 kg/m^2^)	2.38 (0.71, 6.03)	0.103
Obese (> 30 kg/m^2^)	1.24 (0.80, 1.92)	0.333
Ethnic identity (MEIM)	1.39 (0.87, 2.27)	0.176
Discrimination		
None reported	1.00 (Reference)	
At least one report	1.54 (0.89, 2.86)	0.142
Education		
Primary/secondary education	1.00 (Reference)	
Completed tertiary education	0.90 (0.51, 1.51)	0.705
Income		
Below LICO	1.00 (Reference)	
Above before-tax LICO	0.90 (0.48, 1.57)	0.717

LICO refers to the low-income cutoff as defined by Statistics Canada. *MEIM*, Multi-Ethnic Identity Measure (Phinney, 1992)

After examining bivariate relationships and discussing the likely experiences of the target population, we excluded exercise, smoking and housing from our multivariable model. Exercise was excluded because the majority of participants, both with and without CVD, reported exercising 7 days a week, and there was no observed reduction in CVD risk associated with exercise (RR = 0.99; 95% CI: 0.92, 1.08). We excluded smoking because our community partners and medical experts recognized that a diagnosis of CVD may have contributed to quitting for this sample and that in the OHC sample, 68% were current smokers. Furthermore, current smokers were *less likely* to have CVD than non-smokers. A reasonable assumption is that we are observing the ‘sick-quitter’ effect (Shaper et al., 1988) where, for some, a diagnosis was incentive to quit. The housing variable was similar in that we found those reporting homelessness had lower CVD prevalence than people who were stably housed. Experiencing homeless with CVD may have led to housing supports or institutionalization, and, as a result, CVD may be directly influencing housing.

### Refined multivariable model

Variables were removed from the preliminary model in the following order: education, income, gender and ethnic identity (MEIM score). At each deletion, the AIC statistic was reduced and the predictive statistics either remained unchanged or improved slightly and the relative risks of the remaining variables remained stable, indicating that no unique information was being lost. Removing these variables enables more precise estimation of the effects of the variables that remain in the model. The final model is presented in Table 4.

**Table 4:** Final model of cardiovascular disease risk factors. Relative risks are presented for both the development sample (Toronto, *n*=862) and the validation sample (Hamilton, *n*=531)

	Toronto (development sample)		Hamilton (validation sample)	
Variable	RR (95% CI)	*p* value	RR (95% CI)	*p* value
Age	1.05 (1.04, 1.07)	<0.001	1.04 (1.02, 1.06)	<0.001
Diabetes and hypertension				
Neither condition	1.00 (Reference)		1.00 (Reference)	
Diabetes	1.66 (0.73, 3.46)	0.196	2.98 (1.23, 7.28)	0.016
Hypertension	2.98 (1.74, 5.12)	<0.001	2.62 (1.36, 5.08)	0.004
Both conditions	3.03 (1.66, 5.50)	<0.001	4.38 (2.20, 8.71)	<0.001
Body mass index (BMI)				
Healthy or overweight	1.00 (Reference)		1.00 (Reference)	
Underweight	2.72 (0.82, 6.72)	0.056	insufficient data	
Obese	1.30 (0.85, 2.00)	0.225	0.95 (0.56, 1.60)	0.843
Discrimination				
No	1.00 (Reference)		1.00 (Reference)	
Yes	1.53 (0.89. 2.80)	0.143	2.10 (1.13, 3.89)	0.019

### Model validation

**Fig. 1:**
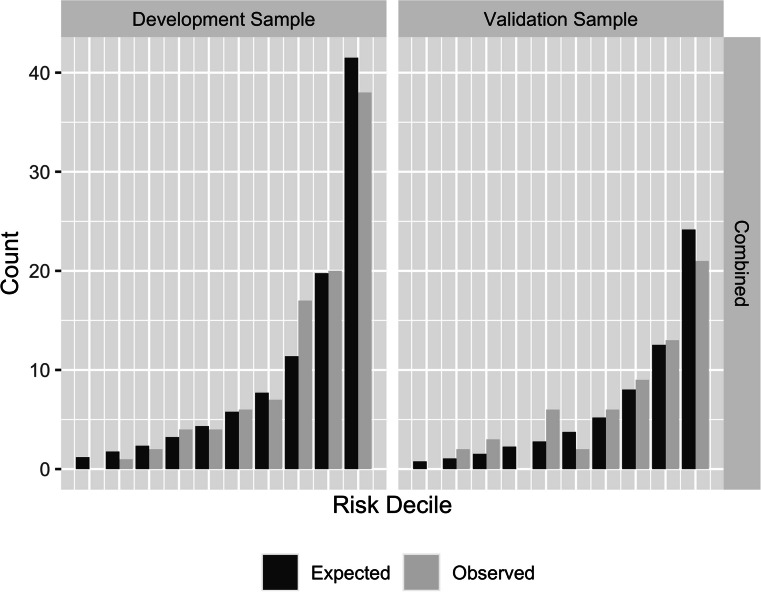
Prediction of CVD prevalence for the model development sample (Toronto) and validation sample (Hamilton). Hamilton predictions have been adjusted to account for different overall prevalence in the populations

**Fig. 2:**
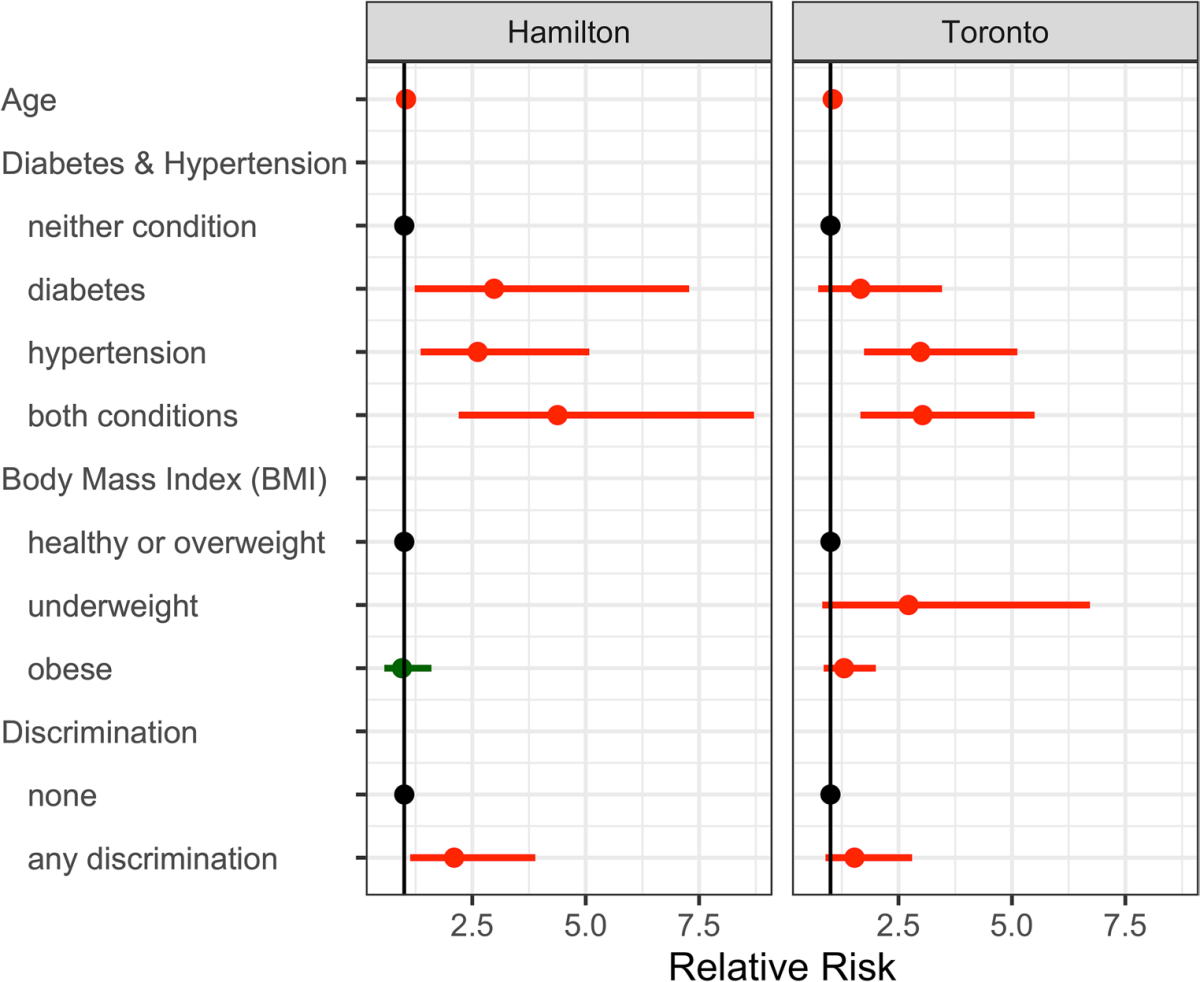
Relative risk associated with cardiovascular disease among the urban Indigenous community living in Toronto and Hamilton

Table 5 contains the measures of model validation for the Toronto and Hamilton samples. To adjust the baseline prevalence for differences in the Toronto and Hamilton samples, a conversion factor of *cf* = 0.22 was calculated and used to adjust the model discrimination values. No adjustment was needed for the *c*-index, being a rank-based statistic. Figure 1 shows the actual and model-predicted CVD prevalence for each risk decile. In both samples, the observed counts are similar to the model predictions with an overestimate of prevalence in the highest decile. The measures of model calibration and discrimination are model-level indices and do not provide information on the performance of individual predictors. To better evaluate the performance of the model in a new sample, an unweighted Poisson model was fit to the Hamilton data. These results are presented in Table 4 and graphically in Fig. 2.

**Table 5:** Model discrimination (*c-*index), calibration (Hosmer and Lemeshow *χ*^2^) and predictive statistics for the model development and validation samples

Model	Development sample	Validation sample
Sample size	862	531
*c*-index	0.83	0.79
Model calibration - adjusted		
Hosmer and Lemeshow GOF *χ*^2^	5.65	11.3
*p* value	0.686	0.184
Model calibration - unadjusted		
Hosmer and Lemeshow GOF *χ*^2^		17.0
*p* value		0.030
Model predictive statistics		
Sensitivity	0.23	0.10
Specificity	0.99	0.99
Positive predictive value	0.68	0.50
Negative predictive value	0.90	0.89
Accuracy	0.90	0.88

## Discussion

Using a transparent, in-depth modelling approach in which the knowledge of community health professionals was prioritized, along with statistical results, we developed and validated a model of factors associated with the prevalence of CVD in the Indigenous community living in Toronto. We began with a list of factors we knew or hypothesized, based on the literature and/or Indigenous health expertise, would be associated with CVD: age, gender, BMI, diabetes, hypertension, cigarette smoking, exercise, education, income, housing, Indigenous self-identity and experiences of discrimination. With the exception of age, which we accepted as fundamental, we considered each variable and whether it warranted inclusion in a multivariable model based on existing scholarship and the collective expertise of the research team. Age-adjusted models of risk and data visualization were used to explore potential interactions. For variables expected to predict prevalent CVD, we fit an initial multivariable model. We then sought to make the model more parsimonious by removing variables that did not improve the fit or predictive ability of the model. The final model was validated using a similar sample of Indigenous peoples living in Hamilton, Ontario. We have shed light on the ‘black-box’ often used in model building, exposing our variable inclusion process (expounded upon in the Supplemental Information) and our initial multivariable model. This approach is expected to assist in better understanding the complex dynamics involved in fitting a CVD model from cross-sectional data. In particular, we highlight the importance of using community-based knowledges and information to ensure a culturally relevant model as well as the need to consider the possibility of disease status modifying exposure. The crucial step of model validation was also demonstrated, providing evidence that the model is replicable, that the modelling process did not over-fit the data and that, despite differences between the populations (including land custodianship and size), the results are applicable to a distinct population with similar eco-social systems.

The final risk model for CVD (Table 4) included age, a combined variable of diabetes and hypertension, categorized BMI, and a dichotomous variable that was coded ‘yes’ if participants had experienced discrimination on any of the topics included in the survey questions, including discrimination from a healthcare provider, because of Indigenous identity, because of a health problem or because of emotional or mental problems (full descriptions provided in Table 2). The results were in line with expectations: age was the most significant predictor of CVD, increasing 5% each year as was found in the work validating the Framingham scores (D’Agostino et al., 2001). Diabetes and hypertension were predictive of CVD, and in combination produced the greatest risk; Ohishi (2018) explains that diabetes causes vascular remodelling which contributes to hypertension among those with mid-stage disease and noted a six-fold increase for those with both conditions among a Japanese cohort. Our findings indicated a three-fold increase in the Toronto sample and a four-fold increase in the Hamilton sample which may be explained by a difference in the diabetic stage of the participants; in the Toronto sample, diabetes was often associated with hypertension. When we examined the bivariate relationship between BMI and CVD, we did not identify a significant difference in risk associated with being overweight and used those in the normal to overweight range as the reference group. Obese individuals (BMI > 30 kg/m^2^) and underweight individuals (BMI < 18.5 kg/m^2^) were more likely to have CVD relative to those who were healthy weight or overweight. These findings should be interpreted cautiously due to the large confidence intervals. However, the findings are consistent with findings from Bogers et al. (2007) and Park et al. (2017) who reported a j-shaped relationship between BMI and CVD, with deviations from normal BMI (18.5–25 kg/m^2^) associated with increased risk.

Discrimination increased the risk of CVD by over 50% in the OHC Toronto sample and by a factor of more than two in the OHC Hamilton sample. We note that discrimination was not statistically significant but was nevertheless retained in the final model. This was based on a plausible biological pathway (discrimination leading to stress and other CVD-related outcomes), the large point estimate of the effect size in a bivariate analysis and ambiguity in the literature. Furthermore, discrimination is associated with a broad range of poor health outcomes among Indigenous peoples, including commercial tobacco use, blood pressure, heart disease and diabetes, and is a fundamental determinant of health and health inequities (Thurber et al.,^ 2021^). This finding has important implications for healthcare delivery. Evidence has been mounting regarding the association between discrimination and CVD. In their survey of discrimination and CVD, Lewis et al. (2014) found heterogeneity across studies, which they attributed to the difficulties in measuring discrimination and in modelling the complex pathways linking discrimination and CVD. Chae et al. (2010) investigated these complexities and found support for the hypothesis that stress is the mechanism by which discrimination adversely affects cardiovascular health, and the World Health Organization recognizes social exclusion, including discrimination, as a driver of poor health and CVD in particular (Wilkinson & Marmot, 2003). A recent study by Currie et al. (2020) found a linear relationship between the number of experiences of discrimination within the previous year and allostatic load, a measure of burden of physiological stress. This provides further evidence for the mediating role of stress in the pathway between discrimination and CVD. What remains unknown is the nature of the pathways linking discrimination, stress, hypertension, diabetes and CVD. The current study defined discrimination as a dichotomous variable, instead of creating a summary score. This was done to enhance interpretability as the resulting estimate is the increased risk associated with exposure to *any* discrimination. However, we are unable to tease out the contributions of varying forms of discrimination, nor can we determine how cumulative exposure contributes to risk. Thus, while further study is required to determine whether various sources of discrimination have differential impacts on health, there is a clear rationale for reducing exposure to discrimination among Indigenous peoples.

The OHC study collected information about gender but not sex. Women were found to have slightly higher risk of CVD than men, but the confidence intervals around the risk estimate were wide and removing gender was not detrimental to the fit nor predictive ability of the model. Exploratory analyses of gender and BMI interactions were inconclusive. Our results contrasted with several other studies (Anand et al., 2001; Park et al., 2017), including those on Indigenous populations (Anand et al., 2006) which found higher rates of CVD for men. However, Dannenbaum et al. (2008) found the prevalence of diabetes among Cree living in Eeyou Istchee in 2005 was higher for females than for males in all age groups. The role of sex and gender in the development of diabetes and CVD among Indigenous peoples in Canada is unclear and warrants further study.

Our preliminary multivariable model (Table 3) indicated, as expected, that income above the low-income cutoff and a tertiary qualification were preventive with respect to CVD. However, effect sizes were small, with wide confidence intervals for both variables. We suspect a larger sample or one more homogenous with respect to age and comorbidities is necessary to provide conclusive evidence regarding the role of education and income in this population. Indigenous identity, as measured by the MEIM, was positively associated with CVD. This result contrasted with our expectations and was intriguing; we hypothesized it might have reflected differential treatment of Indigenous peoples based on their outward expression of Indigenous identity or may simply have been an artifact of the data.

Our final model was 90% accurate in predicting self-reported CVD in the OHC Toronto (development) sample and 88% accurate for the OHC Hamilton (validation) sample. This high overall accuracy is the result of correctly identifying healthy individuals (specificity) rather than an ability to correctly predict those with CVD. This indicates that our model is incomplete and there are other important correlates of CVD that were not captured in our model. A possible reason for this is the exclusion of smoking and exercise from the model. The participants living in Toronto reported high rates of commercial tobacco use (68%). However, in this sample, current smoking rates were not associated with increased CVD risk. We suspect that, had information about former smoking behaviours been available, it would have made an important contribution to the model. Despite the low sensitivity of the model, we are confident that our findings are generalizable to other similar populations of Indigenous peoples living in urban areas. The model has relatively high discriminative ability, good calibration ability and, most importantly, similar findings of risk across distinct and independent samples. In both samples, diabetes, hypertension and prior experience of discrimination were associated with increased risk of CVD. The relationship between BMI and CVD in these populations is unclear and further work to determine the extent to which body size affects CVD among Indigenous peoples is required.

Road maps for effecting positive change in Indigenous health already exist. The Truth and Reconciliation Commission of Canada (TRC) has outlined 94 calls to action (TRC, 2015), which, if implemented, will reduce health inequities between Indigenous and non-Indigenous peoples. Reducing discrimination is specifically targeted by calls to implement skills-based training in intercultural competency for law, nursing and medical students and professionals. At a systems level, the TRC recommended the following: recognition that current-day Indigenous/non-Indigenous health inequities are rooted in harmful governmental polices; establishment of measurable health equity goals and tracking progress; provision of sustainable funding for expanded Indigenous healing centres; recognition and use of Indigenous healing practices; and increasing the number of Indigenous health professionals. Calls to reduce inequities in education and child welfare and to incorporate Indigenous content into school curriculums will also foster social inclusion and reduce inequities. Along with addressing the TRC calls to action, Canada and its provinces should adopt the Health in All Policies (HiAP) framework, which can be implemented with established toolkits (Tonelli et al., 2020). HiAP approaches—which require health to be considered in all areas of government—resonate with Indigenous approaches to health through the shared acknowledgement that the roots of health are broad and cross-sectoral. Federal and provincial governments need to embrace HiAP and fully action the TRC’s calls. The media can be a vehicle for governmental accountability, as demonstrated by Beyond 94 from the CBC (https://newsinteractives.cbc.ca). Ongoing community leadership is essential.

Strengths of this study were Indigenous governance, leadership, engagement and participation and model validation using an independent data set. Strengths and limitations of our study included the use of self-reported survey data, consistent with United Nations Declaration on the Rights of Indigenous Peoples (UNDRIP), and undertaking a secondary analysis of data intended to measure the baseline health of a population. By using an existing data set, we were limited in the scope of our analysis to those indicators collected by the survey and, for example, did not have access to smoking history. Respondent-driven sampling, as opposed to probability sampling, is also a limitation, but is the best method available in the absence of an inclusive clinical registry of Indigenous people living in urban and related homelands. Despite this, we have identified discrimination along with hypertension and diabetes as modifiable exposures that could be addressed to improve cardiovascular health. Our validation work provides unique evidence for generalizing these findings to other urban Indigenous communities.

## Contributions to knowledge

What does this study add to existing knowledge?

- Discrimination is a risk factor for cardiovascular disease in Indigenous peoples living in urban centres.
- The relationship between BMI and cardiovascular disease is unclear. Further research into whether being underweight is a risk factor for cardiovascular disease is required.
- Careful consideration of causal mechanisms is essential to model development. To prevent over-fitting and spurious results arising from a data-driven approach requires model validation on independent samples.

What are the key implications for public health interventions, practice or policy?

- Consistent with the Truth and Reconciliation Commission, eliminating discrimination is urgently required as well as policy and clinical practice to improve health outcomes, including cardiovascular health.

## Supplementary information

ESM 1 (DOCX 25 kb)
