# Supplementary material for: Modelling prevalent cardiovascular disease in an urban Indigenous population
Source: Can J Public Health. 2022 Aug 9;115(Suppl 2):288–300. doi: 10.17269/s41997-022-00669-x (PMC11582272; doi:10.17269/s41997-022-00669-x)
Supplement: Supplementary file 1 — (DOCX 25 kb) [file 41997_2022_669_MOESM1_ESM.docx]

# Supplemental Information

## 1.1 Outcome

An affirmative answer to either of the following questions was considered prevalence CVD: *Have you been told by a healthcare provider that you have any of the following chronic health conditions: Stroke or Heart Disease*.

## 1.2 Body size

Overweight and obesity are well-established risk factors for CVD, but the best approach for modelling BMI is unclear. A j-shaped relationship has been reported, with both underweight and overweight/obese individuals at increased risk relative to those of normal weight (Bogers et al., 2007; Park et al., 2017). Body weight and height were measured in the study and body mass index (BMI) was calculated. To determine whether BMI should be treated as a continuous predictor we examined plots of CVD prevalence across commonly considered ordinal levels of BMI: underweight (<18.5) healthy weight (18.5-25) overweight (>25), obese I (>30) obese II (> 35) and obese III (>40). Plots indicated that, relative to people with healthy weight, both underweight and obese individuals had higher CVD prevalence, but overweight people had slightly lower prevalence, this was confirmed with a Poisson regression which controlled for age. Based on these findings we chose to model BMI as a categorical variable with healthy/overweight forming the reference category, underweight as a distinct group and all levels of obesity grouped together.

## 1.3 Diabetes and Hypertension

Ohishi described a complicated relationship among hypertension, diabetes, obesity and CVD in which the risk of CVD is greater if both conditions are present than for either condition alone (Ohishi, 2018). We were limited in our analysis to self-reported hypertension and diabetes. To ensure a model that reflected our knowledge of the biological mechanisms contributing to CVD we created a combined variable which classified participants into four groups: 1) those reporting neither condition, 2) those with diabetes alone, 3) hypertension alone or 4) those with both conditions. Mathematically this approach is identical to modelling an interaction term between diabetes and hypertension, but we chose this approach because it provides a very clear interpretation of the risk associated with the individual and combined conditions.

## 1.4 Cigarette Smoking

Smoking is an important risk factor for CVD, even in small quantities. The US Surgeon General has linked commercial tobacco use and exposure to CVD (CDC 2014) and Hackshaw et al. (Hackshaw et al., 2018) reported a non-linear relationship between risk and number of cigarettes smoked, with a high level of risk associated with minimal exposure (1 cigarette per day). In the OHC sample the majority of the sample (68%) were current smokers, consistent with previous reports of higher commercial tobacco use among Indigenous populations (Maddox et al 2018) and were less likely to have CVD than non-smokers. We suspect that lower smoking rates among those with diagnosed CVD may be a result of smoking cessation subsequent to a CVD diagnosis. Because smoking behaviour may be affected by a diagnosis of CVD and because no data is available regarding smoking history we excluded smoking in our multivariable model.

## 1.5 Exercise

The role of exercise in cardiovascular health is well established (Nystoriak & Bhatnagar, 2018). In the OHC Toronto sample, the self-reported number of days a week spent exercising was very similar for both those with and without CVD and level of exercise was high, with the majority of participants in both groups reported exercising seven days a week. After adjusting for age, there was no association between days of exercise per week and CVD (RR = 0.99, 95% CI 0.92, 1.08) so exercise was not included in our multivariable model.

## 1.6 Education

Education is a social determinant of health, with increased education associated with several modifiable risk factors, and with lower CVD incidence in several large-scale prospective studies (D’egano et al., 2017; M’ejean et al., 2013; Silventoinen et al., 2005; Van Lenthe et al., 2002). The effect of education on incidence of CVD has been partially explained by biological factors. These include metabolic syndrome, diabetes, BMI and hypertension (D’egano et al., 2017; Silventoinen et al., 2005), and more strongly explained by smoking, diet and alcohol consumption (M’ejean et al., 2013; Van Lenthe et al., 2002). After controlling for age, relative to those who have a primary level of education, completion of a tertiary qualification was associated with a small, non-significant reduction in CVD prevalence (RR = 0.86, 95% CI 0.50, 1.42) and completion of high school was not associated with a reduced risk (RR = 1.00, 95% CI 0.64, 1.53). We therefore collapsed the lower two educational levels (primary/secondary) to explore the impact of completion of a tertiary qualification on CVD prevalence. We classified tertiary education as all those who completed university, college or specialized trades training and included this in our multivariable model.

## 1.7 Income

Income is a social determinant of health with rates of CVD increasing with declining income (Kreatsoulas & Anand, 2010). This is particularly important given the income disparity between Indigenous and non-Indigenous people and its link to excess mortality from CVD (Tjepkema et al., 2012). Data on total household income and household size were collected and used, along with tables provided by Statistics Canada (Income Statistics Division, 2015) to dichotomise participants into those above and below the before tax low income cut-off (LICO). The LICO threshold indicates the threshold ‘below which a family will likely devote a larger share of its income on the necessities of food, shelter and clothing than the average family’. Because the LICO adjusts for average family earnings in the same geographic area, as well as family size we viewed it as a more informative indicator of financial security than total income. Being above the LICO carried a non-significant reduced risk of CVD (RR = 0.80; 95% CI 0.45, 1.32) and was included in the multivariable model.

## 1.8 Multi-Ethnic Identity Measure MEIM

The multi-ethnic identity measure (MEIM) (Phinney, 1992), was used to assess feelings of affirmation and belonging and ethnic identity. We used the total MEIM score, which is comprised of twelve items measured on a four-point Likert scale, with higher total score indicating stronger ethnic identity. The measure demonstrated good validity among a sample of college students (Cronbach’s $\alpha$ = 0.90) (Phinney, 1992). Increased ethnic identity was associated with a 70% *higher* risk of CVD (RR = 1.72; 95% CI 1.14, 2.63), in contrast to our *a priori* expectation that strong ethnic identity would be protective. We hypothesized that our observation of increased CVD for those with stronger ethnic identities may be linked to differential treatment of Indigenous peoples based on their identities. The MEIM was included in the multivariable model because a diagnosis of CVD would not have affected ethnic identity and we could theorise of a mechanism by which strong identity influenced CVD mediated by discrimination.

## 1.9 Discrimination

Discrimination was scored dichotomously with an experience of discrimination recorded if participants reported any type of unfair treatment including: 1) unfair treatment because of being Indigenous, 2) unfair treatment because of mental or emotional problems, 3) unfair treatment because of gender or 4) unfair treatment by a healthcare worker. Discrimination was associated with a 60% increase in risk for CVD, although the confidence intervals were wide (RR = 1.61; 95% CI 0.97, 2.86); it was included in the multivariable model.

## 1.10 Housing

Participants were categorised into four levels of housing stability: 1) stable housing (having a permanent place to stay), 2) precarious housing (staying with friends or relatives or in a motel, 3) institutionalised (nursing home or hospital) or 4) homeless. Relative to those in stable housing, those in precarious housing had no evidence of a statistically increased risk of CVD (RR = 1.14, 95% CI 0.56, 2.10), the homeless were only half as likely to report CVD (RR = 0.51, 95% CI 0.23, 1.00) and there was too little data to draw conclusions about institutionalised persons (RR = 4.16, 95% CI 0.24, 18.7). We decided that housing was a poor predictor of prevalent CVD because those who are ill are more likely to be institutionalised and less likely to be unstably housed; thus this variable was not included in our multivariable model.

## 1.11 Sex/Gender

The role of sex/gender in CVD is complicated enough that many studies (including Framingham) model risk separately for males and females. Traditionally, rates of CVD are higher among males/men than females/women, as are rates of metabolic disorders. However, rates among females/women are increasing more quickly than among males/men (Regitz-Zagrosek et al., 2006). OHC did not collect information about biological sex, and the sample is not large enough to stratify by sex/gender. We investigated the relationship between cis-gendered individuals and CVD and found a non-significant age-adjusted increased risk of CVD for females relative to males (RR = 1.35; 95% CI 0.91, 2.04). Relationships between sex/gender and both body size and diabetes/hypertension were examined graphically. As a result, we did not model interaction terms, but did include gender for consideration in the multivariable model.

# References

Bogers, R. P., Bemelmans, W. J. E., Hoogenveen, R. T., Boshuizen, H. C., Woodward, M., Knekt, P., Van Dam, R. M., Hu, F. B., Visscher, T. L. S., Menotti, A., Thorpe, R. J., Jamrozik, K., Calling, S., Strand, B. H., & Shipley, M. J. (2007). Association of overweight with increased risk of coronary heart disease partly independent of blood pressure and cholesterol levels: A meta-analysis of 21 cohort studies including more than 300 000 persons. *Archives of Internal Medicine*, *167*(16), 1720–1728. <https://doi.org/10.1001/archinte.167.16.1720>

Centers for Disease Control and Prevention, Department of Health and Human Services, National Center for Chronic Disease Prevention and Health Promotion, Office on Smoking and Health, 2014. The health consequences of smoking – 50 years of progress: a report of the Surgeon General. – Atlanta, GA. : U.S.

Dégano, I. R., Marrugat, J., Grau, M., Salvador-González, B., Ramos, R., Zamora, A., Martí, R., & Elosua, R. (2017). The association between education and cardiovascular disease incidence is mediated by hypertension, diabetes, and body mass index. *Scientific Reports*, *7*(1), 1–8. <https://doi.org/10.1038/s41598-017-10775-3>

Hackshaw, A., Morris, J. K., Boniface, S., Tang, J. L., & Milenkovi, D. (2018). Low cigarette consumption and risk of coronary heart disease and stroke: Meta-analysis of 141 cohort studies in 55 study reports. *BMJ (Online)*, *360*. <https://doi.org/10.1136/bmj.j5855>

Income Statistics Division. (2015). *Low Income Lines , 2013-2014* (Nos. 75; p. 39). Statistics Canada. <http://www.statcan.gc.ca/pub/75f0002m/75f0002m2011002-eng.pdf>

Kreatsoulas, C., & Anand, S. S. (2010). The impact of social determinants on cardiovascular disease. *Canadian Journal of Cardiology*, *26*, 8C–13C. <https://doi.org/10.1016/S0828-282X(10)71075-8>

Maddox R, Waa A, Lee K, Nez Henderson P, Blais G, Reading J and Lovett, R (2018). Commercial tobacco and Indigenous peoples: a stock take on Framework Convention on Tobacco Control progress. Tobacco Control.

Méjean, C., Droomers, M., Van Der Schouw, Y. T., Sluijs, I., Czernichow, S., Grobbee, D. E., Bueno-De-Mesquita, H. B., & Beulens, J. W. J. (2013). The contribution of diet and lifestyle to socioeconomic inequalities in cardiovascular morbidity and mortality. *International Journal of Cardiology*, *168*(6), 5190–5195. <https://doi.org/10.1016/j.ijcard.2013.07.188>

Nystoriak, M. A., & Bhatnagar, A. (2018). Cardiovascular Effects and Benefits of Exercise. *Frontiers in Cardiovascular Medicine*, *5*(September), 1–11. <https://doi.org/10.3389/fcvm.2018.00135>

Ohishi, M. (2018). Hypertension with diabetes mellitus: Physiology and pathology review-article. *Hypertension Research*, *41*(6), 389–393. <https://doi.org/10.1038/s41440-018-0034-4>

Park, D., Lee, J.-H., & Han, S. (2017). Underweight: another risk factor for cardiovascular disease? A cross-sectional 2013 Behavioral Risk Factor Surveillance System (BRFSS) study of 491,773 individuals in the USA. *BRFSS) of Centers for Disease Control and Prevention*, *48*(October). <https://doi.org/10.1097/MD.0000000000008769>

Phinney, J. (1992). The Multigroup Ethnic Identity Measure. *Journal of Adolescent Research*, *7*(2), 156–176.

Regitz-Zagrosek, V., Lehmkuhl, E., & Weickert, M. O. (2006). Gender differences in the metabolic syndrome and their role for cardiovascular disease. *Clinical Research in Cardiology*, *95*(3), 136–147. <https://doi.org/10.1007/s00392-006-0351-5>

Silventoinen, K., Pankow, J., Jousilahti, P., Hu, G., & Toumilehto, J. (2005). Educational inequalities in the metabolic syndrome and coronary heart disease among middle-aged men and women. *International Journal of Epidemiology*, *34*(2), 327–334. <https://doi.org/10.1093/ije/dyi007>

Tjepkema, M., Wilkins, R., Goedhuis, N., & Pennock, J. (2012). Cardiovascular disease mortality among first nations people in Canada, 1991-2001. *Chronic Diseases and Injuries in Canada*, *32*(4), 200–207.

Van Lenthe, F. J., Gevers, E., Joung, I. M. A., Bosma, H., & Mackenbach, J. P. (2002). Material and behavioral factors in the explanation of educational differences in incidence of acute myocardial infarction: The globe study. *Annals of Epidemiology*, *12*(8), 535–542. <https://doi.org/10.1016/S1047-2797(01)00279-4>
